# Enhanced survival of *Leishmania major* in neutrophil granulocytes in the presence of apoptotic cells

**DOI:** 10.1371/journal.pone.0171850

**Published:** 2017-02-10

**Authors:** Natallia Salei, Lars Hellberg, Jörg Köhl, Tamás Laskay

**Affiliations:** 1 Department of Infectious Diseases and Microbiology, University of Lübeck, Lübeck, Germany; 2 Institute for Systemic Inflammation Research, University of Lübeck, Lübeck, Germany; INRS—Institut Armand Frappier, CANADA

## Abstract

Neutrophil granulocytes are the first leukocytes that encounter and phagocytose *Leishmania major* (*L*. *major*) parasites in the infected skin. The parasites can nonetheless survive within neutrophils. However, the mechanisms enabling the survival of *Leishmania* within neutrophils are still elusive. Previous findings indicated that human neutrophils can engulf apoptotic cells. Since apoptotic neutrophils are abundant in infected tissues, we hypothesized that the uptake of apoptotic cells results in diminished anti-leishmanial activity and, consequently, contributes to enhanced survival of the parasites at the site of infection. In the present study, we demonstrated that *L*. *major*-infected primary human neutrophils acquire enhanced capacity to engulf apoptotic cells. This was associated with increased expression of the complement receptors 1 and 3 involved in phagocytosis of apoptotic cells. Next, we showed that ingestion of apoptotic cells affects neutrophil antimicrobial functions. We observed that phagocytosis of apoptotic cells by neutrophils downregulates the phosphorylation of p38 MAPK and PKCδ, the kinases involved in activation of NADPH oxidase and hence reactive oxygen species (ROS) production. In line, uptake of apoptotic cells inhibits TNF- and *L*. *major*-induced ROS production by neutrophils. Importantly, we found that the survival of *Leishmania* in neutrophils is strongly enhanced in neutrophils exposed to apoptotic cells. Together, our findings reveal that apoptotic cells promote *L*. *major* survival within neutrophils by downregulating critical antimicrobial functions. This suggests that the induction of enhanced uptake of apoptotic cells represents a novel evasion mechanism of the parasites that facilitates their survival in neutrophil granulocytes.

## Introduction

*Leishmania major* (*L*. *major*) is an obligatory intracellular parasite transmitted by sandflies. *L*. *major* infection leads to massive recruitment of neutrophil granulocytes to the skin where they rapidly internalize the parasites [1]. Although neutrophils are equipped with a deadly array of antimicrobial effector tools, *L*. *major* can still survive inside neutrophils [2]. *Leishmania* parasites possess potent evasion mechanisms that allow their survival and multiplication in macrophages [3]. However, mechanisms critical for their survival in neutrophils still remain elusive. *Leishmania* suppresses reactive oxygen species (ROS) generation in neutrophils [2,4], implying that the parasites may possess potent–but yet poorly characterized–counter-regulatory mechanisms.

Neutrophils are short-living cells and undergo apoptosis within few hours. Many apoptotic neutrophils accumulate at sites of infection [5]. We and others have previously demonstrated that human neutrophils, similarly to macrophages, can engulf apoptotic material [6,7]. It is widely accepted that uptake of apoptotic cells by phagocytes has an anti-inflammatory effect [8,9]. For instance, in vitro studies showed that signals from apoptotic cells diminish the secretion of pro-inflammatory cytokines, such as tumor necrosis factor (TNF), interleukin (IL)-1β and CXCL10, and the generation of ROS by macrophages [10,11] and neutrophils [6], as well as stimulate the release of anti-inflammatory cytokines, such as IL-10 [12]. Contact to apoptotic cells has been shown to compromise antimicrobial defense. For instance, interaction with apoptotic cells increases parasite burden in *Leishmania amazonensis*-infected human macrophages in culture [13]. Therefore, we hypothesized that apoptotic cells promote the survival of *L*. *major* parasites inside neutrophils.

Phagocytosis of apoptotic cells is markedly enhanced in the presence of normal (non-heat inactivated) human serum suggesting the involvement of the complement factors [14]. Previously, it has been shown that serum factors significantly increase the uptake of apoptotic cells by other phagocytes. For instance, the depletion of complement factors C1q and C3 from serum markedly decreases phagocytosis of apoptotic cells by macrophages [14,15]. However, the role of the complement factors in neutrophil-mediated phagocytosis of apoptotic cells has not been addressed so far.

In the present study, we investigate how apoptotic cells affect the survival of *L*. *major* within human neutrophil granulocytes in vitro. We show that infection of neutrophils with *L*. *major* facilitates the uptake of apoptotic cells. In turn, a presence of apoptotic cells leads to diminished ROS production and improved *L*. *major* survival within neutrophils. These findings support the view that *L*. *major* facilitates its own survival within neutrophils by modulating the uptake of apoptotic cells.

## Materials and methods

### Isolation of human peripheral blood neutrophil granulocytes

Peripheral blood was collected by venipuncture from healthy adult volunteers in lithium-heparin-containing tubes. All studies as well as consent procedure for blood donors were approved by the ethics committee of the University of Lübeck (05–124). All blood donors provided written agreement to participate in the study. Neutrophils were isolated by discontinuous Percoll gradient centrifugation as described previously [6]. After isolation neutrophils were re-suspended in complete medium, namely RPMI-1640 medium (Sigma-Aldrich, Steinheim, Germany) supplemented with 10% heat-inactivated fetal calf serum (Sigma-Aldrich), 4 mM L-glutamine, 10 mM HEPES, 100 U/ml penicillin and 100 μg/ml streptomycin (all from Biochrom, Berlin, Germany). The cell preparations contained >99.9% granulocytes as determined by morphological examination of cytocentrifuge slides stained with Diff Quik (Medion Diagnostics, Düdingen, Switzerland).

### Generation of apoptotic cells

Apoptotic neutrophils were obtained by irradiation of freshly isolated neutrophils (2 × 10^7^ per ml) with 256-nm wavelength UV light 800 mJ/cm^2^ using a Stratalinker (Stratagene, Heidelberg, Germany) followed by 4-hour incubation in the complete medium at 37°C in humidified atmosphere containing 5% CO_2_. Apoptosis rate was determined by annexin V-FLUOS (Roche Molecular Biologicals, Mannheim, Germany) and propidium iodide (PI; Sigma-Aldrich) staining and analyzed by flow cytometry. Irradiation led to generation of >85% of apoptotic cells (annexin V positive, PI negative).

### *Leishmania* culture

The origin and propagation of the cloned line of *L*. *major* strain, MHOM/IL/8l/FEBNI, has been described previously [16]. Briefly, *L*. *major* promastigotes were cultured on microtiter plates (Sigma-Aldrich) on rabbit blood agar at 26°C in humidified atmosphere containing 5% CO_2_ for 7 days. Each microtiter plate well contained 100 μl complete medium and 50 μl of a Novy-Nicolle-MacNeal (NNN) blood agar slant, which was prepared by supplementing 200 ml of Brain-Heart-lnfusion agar base (Difco, Detroit, MI, USA) with 50 ml defribrinated fresh rabbit blood (Elocin-Lab, Oberhausen, Germany).

### Infection of neutrophils with *L*. *major*

Neutrophils (5 × 10^6^ per ml) were co-incubated with stationary phase *L*. *major* promastigotes in complete medium at neutrophils-parasite ratio 1:10 for 4 hours at 37°C. Subsequently, neutrophils were washed four times (400 × g, 10 min) with PBS in order to remove non-ingested *L*. *major*. The infection rate and parasite load were determined by morphological examination cytocentrifuge slides stained with Diff Quik.

### Phagocytosis of apoptotic cells

The ingestion of apoptotic cells/material was assessed by using flow cytometry as described [6]. Apoptotic neutrophils were labeled with PKH-26 (Sigma-Aldrich) according to the protocol from the manufacturer and used as target cells in the phagocytosis assay. Non-infected and *L*. *major*-infected neutrophils were labeled with the green fluorescent dye PKH-67 (Sigma-Aldrich) and used as effector cells. For phagocytosis, effector neutrophils were co-incubated with apoptotic cells at a ratio of 1 to 4 for 90 min in complete medium supplemented with 30% normal human serum or human sera depleted of C1q (Merck, Darmstadt, Germany) or deficient for C3 (Institute for Systemic Inflammation Research, University of Lübeck, Germany) with or without exogenous C1q (70 μg/ml, Merck) or C3 (70 μg/ml, Institute for Systemic Inflammation Research, University of Lübeck, Germany), respectively, at 37°C in humidified atmosphere containing 5% CO_2_. Phagocytosis was assessed by using the flow cytometer FACS Canto II (BD Biosciences, San Diego, CA, USA) and FlowJo software (FlowJo LLC, Ashland, OR, USA).

### Phagocytosis of latex beads

Non-infected and *L*. *major*-infected neutrophils were co-incubated with latex beads (FluoSpheres^®^ Polystyrene Microspheres, 1.0 μm, yellow-green fluorescent (505/515), Invitrogen, Eugene, OR, USA) at a ratio of 1 to 10 for 30 min in complete medium supplemented with 30% normal human serum at 37°C in humidified atmosphere containing 5% CO_2_. Phagocytosis was assessed by flow cytometry FACS Canto II and FlowJo software.

### Assessment of surface molecule expression

The following antibodies were used for flow cytometry to assess the expression of the corresponding surface proteins: anti-CD11b (PE, Dako, Hamburg, Germany), anti-CD11c (FITC, Biolegend, San Diego, CA, USA), anti-CD14 (PE, Dako), anti-CD35 (FITC, Biolegend), anti-CD44 (FITC, Biolegend). The expression was evaluated using the flow cytometer FACS Canto II and FlowJo software.

### ROS production assay

#### TNF-induced ROS

ROS production was assessed by using a luminol-amplified chemiluminescence assay as described [17,18]. Briefly, neutrophils were incubated without or with apoptotic cells at a ratio 1:2 in the complete medium supplemented with 30% normal human serum for 90 min at 37°C. Neutrophils were then washed with PBS and re-suspended in CL-medium (a custom-made RPMI-1640 medium without phenol red and sodium hydrogen carbonate containing 20 mM HEPES (Biochrom). Cell suspension (2 × 10^6^ per ml) was placed in 96-well Nunclon Delta white microwell plates (Nunc, Langenselbold, Germany) and 0.06 mM luminol (Sigma-Aldrich) was added. ROS production was induced by 100 ng/ml TNF (PeproTech, Rocky Hill, NJ, USA) ROS-dependent chemiluminescence was analyzed using an Infinite 200 reader and the Tecan i-control 1.7 software (Tecan, Crailsheim, Germany). ROS release was monitored for one hour every minute at 37°C. For statistical analysis the area under the curve (AUC) of each sample was calculated. In order to exclude the influence of cell concentration on ROS production, all ROS assays were carried out with identical amounts of cells.

#### *L*. *major*-induced ROS

Neutrophils (2 × 10^6^ per ml) were added in 96-well Nunclon Delta white microwell plates (Nunc, Langenselbold, Germany) and 0.06 mM luminol (Sigma-Aldrich) was added in the absence or presence of apoptotic cells at a ratio of 1:2. ROS production was induced by *L*. *major* at a neutrophils to parasites ratio of 1 to 10. ROS-dependent chemiluminescence was analyzed using an Infinite 200 reader and the Tecan i-control 1.7 software (Tecan). ROS release was monitored for 150 min at 37°C. For statistical analysis the area under the curve (AUC) of each sample was calculated.

### Western blot analysis

Cell lysates were prepared with trichloroacetic acid precipitation as described previously [19], electrophoresed on 10% SDS-PAGE gels (Bio-Rad Laboratories, Hercules, CA, USA), and transferred to nitrocellulose membranes. Membranes were blocked for 30 min at room temperature with Tris-buffered saline with 0.1% Tween^®^ 20 (Serva, Heidelberg, Germany) containing 5% bovine serum albumin (Carl Roth, Karlsruhe, Germany). Then membranes were incubated with primary antibodies (Ab) phospho-p38 MAPK (Thr180/Tyr182) rabbit Ab and phospho-PKCδ (Tyr311) rabbit Ab at 4°C overnight. After washing, membranes were probed with the corresponding secondary Ab (goat anti-rabbit IgG, HRP-linked) for 1 h at room temperature, and then soaked with Immobilon Western chemiluminescent HRP substrate (Millipore, Billerica, MA, USA). Signals were detected by the Fusion FX7 chemiluminescence reader (Vilber Lourmat, Eberhardzell, Germany) and quantified using ImageJ software (NIH, Bethesda, USA). Equal loading of the gel was determined by re-probing membranes with an anti-β-actin rabbit HRP-conjugated Ab. All antibodies were from Cell Signaling Technology (Danvers, MA, USA).

### Quantitation of viable *L*. *major* in neutrophil granulocytes

A limiting dilution culture assay was used to detect viable *L*. *major* parasites in neutrophil granulocytes as described previously [20]. Briefly, serial 1.5-fold dilutions of 100-μl *L*. *major*-infected neutrophil suspension were plated in 12 replicates in 96-well flat bottom microtiter plates containing 50 μl NNN blood agar and 100 μl complete medium. The plates were incubated at 26°C in humidified atmosphere containing 5% CO_2_ for 10 days. The growth of *L*. *major* promastigotes was detected microscopically. The reciprocal of the last dilution resulting in a growth of parasites in >50% of the wells is given as a quantitative measure of the parasite load in the neutrophil cell suspension.

### Statistical analysis

**Fig 1:**
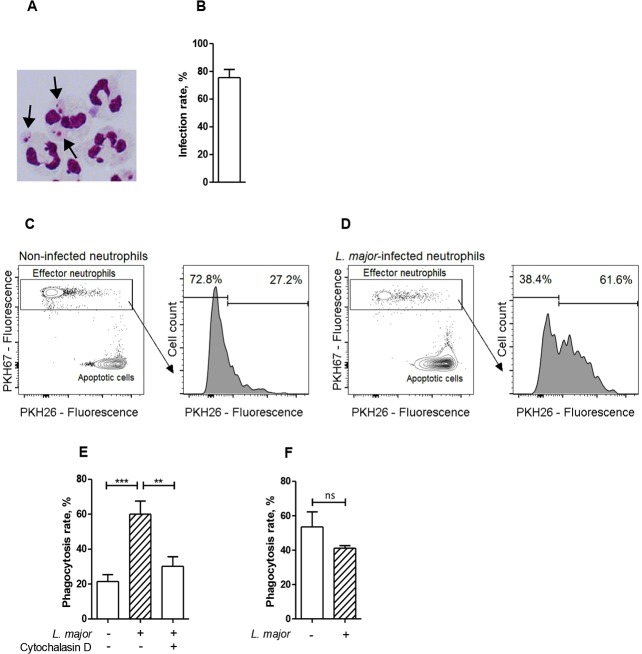
Infection with *Leishmania major* results in enhanced phagocytosis of apoptotic cells by neutrophils. (A) A micrograph of Diff Quik-stained neutrophils infected with *L*. *major* promastigotes. Arrows indicate *L*. *major* parasites inside neutrophils. (B) Percentage of infected neutrophils after co-incubation with *L*. *major* for four hours. (C and D) Representative flow cytometry dot plots and histograms of (C) non-infected and (D) *L*. *major*-infected PKH-67-labeled neutrophils co-incubated with PKH-26-labeled apoptotic cells. (E) Phagocytosis of apoptotic cells by non-infected and *L*. *major*-infected neutrophils in the absence or presence of cytochalasin D. (F) Phagocytosis of latex beads by non-infected and *L*. *major*-infected neutrophils. Data are shown as mean ± SEM of five (B, E) or three (E) independent experiments; *** p ≤ 0.001, ns = not significant.

**Fig 2:**
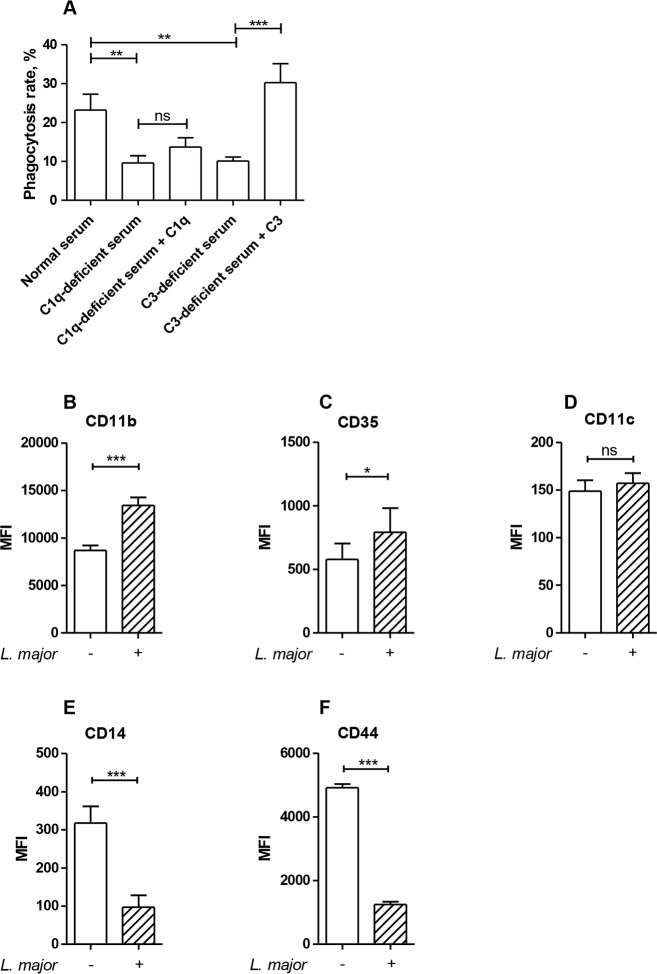
Complement factors are necessary for efficient neutrophil-mediated phagocytosis of apoptotic cells. Neutrophils were co-incubated with apoptotic cells in the presence of 30% normal human serum, C1q-depleted or C3-deficient serum with or without reconstitution of the deficient sera with exogenous C1q or C3, respectively. Phagocytosis of apoptotic cells was assessed by flow cytometry. Data are shown as mean ± SEM of four independent experiments (A). Neutrophils were infected with *L*. *major* promastigotes (ratio of 1 to 10) for 4 h at 37°C. Surface expression of CD11b (B), CD35 (C), CD11c (D), CD14 (E), and CD44 (F) was assessed by flow cytometry. Data are shown as mean ± SEM of three to seven independent experiments. * p ≤ 0.05, ** p ≤ 0.01, *** p ≤ 0.001, ns = not significant.

Comparisons between two groups were performed using Student’s t test for paired samples in GraphPad Prism 5 software (La Jolla, CA USA). For multiple comparisons (Fig 1D and Fig 2A), one-way ANOVA with Bonferroni post-hoc test was used. Differences with a p value < 0.05 or lower were considered significant.

## Results

### Infection with *Leishmania major* promotes phagocytosis of apoptotic cells by neutrophils

To investigate the influence of apoptotic cells on the survival of *L*. *major* in neutrophil granulocytes we first addressed the question whether *Leishmania*-infected neutrophils are able to ingest apoptotic neutrophils. To establish infection, primary human neutrophils were co-incubated with *L*. *major* promastigotes for four hours (Fig 1A and 1B). Next, green PKH67-labeled *Leishmania*-infected and non-infected neutrophils were co-incubated with red PKH26-labeled apoptotic cells and ingestion of the apoptotic material was assessed by flow cytometry. Neutrophils that ingested apoptotic cells acquired red fluorescence (Fig 1C and 1D, S1 Fig and S2 Fig). *L*. *major*-infected neutrophils showed a significantly enhanced capacity to ingest apoptotic cells, compared to non-infected cells (Fig 1E). Moreover, treatment with cytochalasin D, a potent inhibitor of actin polymerization and, therefore, phagocytosis, significantly decreases *L*. *major*-induced phagocytosis of apoptotic cells by neutrophils (Fig 1E), suggesting that apoptotic cells indeed engulfed by neutrophils but not merely adhered. Importantly, *Leishmania* infection had no influence on phagocytosis of other targets, such as latex beads (Fig 1F), implying a specific effect on uptake of apoptotic cells, rather than a general enhancement of the phagocytic capacity of neutrophils induced by *Leishmania* infection.

### *L*. *major*-infected neutrophils upregulate surface expression of receptors involved in the phagocytosis of apoptotic cells

Components of the complement system are likely to be important for the uptake of apoptotic cells by neutrophils, since efficient neutrophil-mediated ingestion of apoptotic cells requires the presence of normal (non-heat inactivated) human serum [6]. The contribution of complement factors was tested by using sera lacking C1q and C3. We observed that ingestion of apoptotic cells by neutrophils was significantly reduced in the presence of C1q- and C3-deficient sera, as compared to the ingestion level in the presence of normal serum (Fig 2A). The phagocytosis rate was completely restored by reconstitution of the C3-deficient serum with exogenous C3, whereas reconstitution of the C1q-depleted serum with exogenous C1q had only minor, insignificant effect (Fig 2A).

Several cell surface receptors are involved in recognition and engulfment of apoptotic cells by phagocytes [21]. The complement receptors CR1 and CR3 were shown to mediate recognition and ingestion of apoptotic cells by dendritic cells and macrophages, whereas a role of CR4 seems less critical [14,22]. Since C1q and C3 contribute to the uptake of apoptotic cells by neutrophils (Fig 2A), we next assessed the cell surface expression of CD35 (CR1) and CD11b (a component of CR3) on non-infected versus *Leishmania*-infected neutrophils. We found that both CD11b and CD35 expression was upregulated on *L*. *major*-infected neutrophils, compared to non-infected cells (Fig 2B and 2C), whereas CD11c (a component of CR4) expression remained unchanged (Fig 2D). On the other hand, the expression of receptors involved in uptake of apoptotic cells by macrophages in a complement-independent manner, such as CD14 and CD44 [23,24], was not elevated, but rather significantly reduced (Fig 2E and 2F). These findings support the view that infection-induced upregulation of CR1 and CR3 may contribute to the enhanced capacity of *Leishmania*-infected neutrophils to engulf apoptotic cells.

### Enhanced survival of *L*. *major* in neutrophils in the presence of apoptotic cells

**Fig 3:**
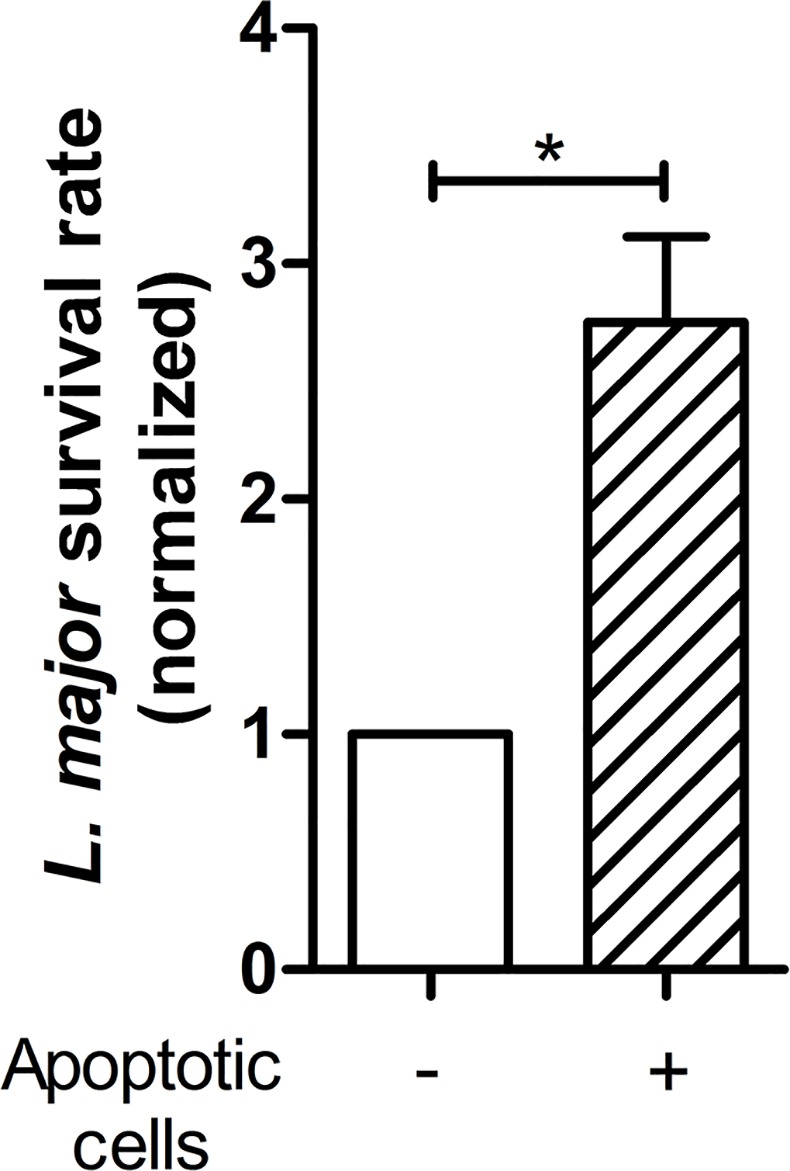
Enhanced survival of *L*. *major* in neutrophils in the presence of apoptotic cells. Neutrophils were infected with *L*. *major* promastigotes (ratio of 1 to 10) for 4 h at 37°C. Then infected neutrophils were incubated in the absence or presence of apoptotic neutrophils for 18 h in culture medium containing normal human serum. Survival of *L*. *major* within neutrophils was assessed by the limiting dilution assay and normalized to *Leishmania* survival rate in neutrophils incubated without apoptotic cells. Data are shown as mean ± SEM of four independent experiments; * p ≤ 0.05.

Interaction with apoptotic cells has been reported to diminish antimicrobial functions of macrophages [13]. Therefore, we hypothesized that contact to apoptotic cells results in diminished antimicrobial functions also in neutrophils and, in turn, leads to enhanced *Leishmania* survival in neutrophils. To test this idea, *L*. *major*-infected neutrophils were cultured for 18 hours in the presence or absence of apoptotic cells, and the numbers of viable parasites were then detected by a limiting dilution culture assay. Infected neutrophils upon co-incubation with apoptotic cells harbored significantly more viable parasites, compared to *Leishmania*-infected neutrophils incubated without apoptotic cells (Fig 3). This finding indicates that exposure of neutrophils to apoptotic material markedly enhances intracellular survival of *Leishmania* parasites.

### Reduced activation of antimicrobial signaling pathways in neutrophils in the presence of apoptotic cells

**Fig 4:**
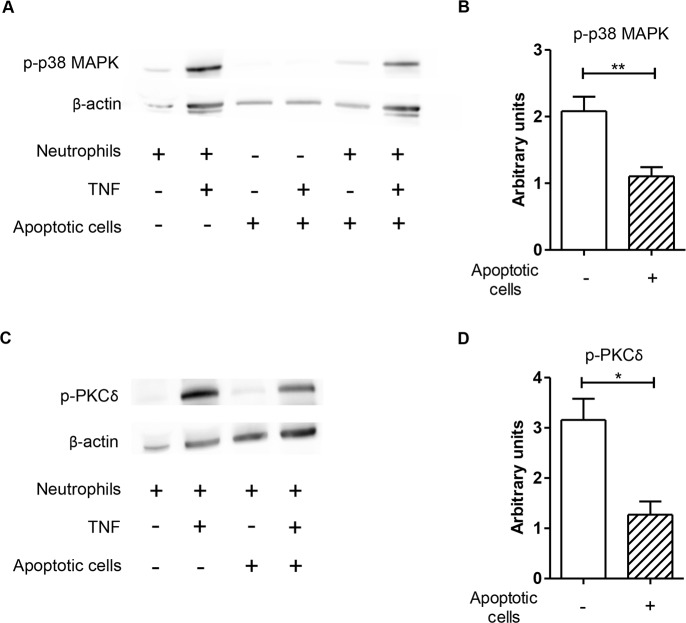
Co-incubation with apoptotic cells results in diminished TNF-induced phosphorylation of PKCδ and p38-MAPK in neutrophils. Freshly isolated neutrophils were incubated in the absence or presence of apoptotic cells for 90 min prior to stimulation with TNF-α for 15 min, and the phosphorylation of p38-MAPK and PKCδ was assessed by western blot. A representative blot of phosphorylated p38-MAPK (A) and PKCδ (C) are shown. Data were quantified and normalized to β-actin (B and D). Data are shown as mean ± SEM of three-four independent experiments; *p ≤ 0.05, **p ≤ 0.01.

**Fig 5:**
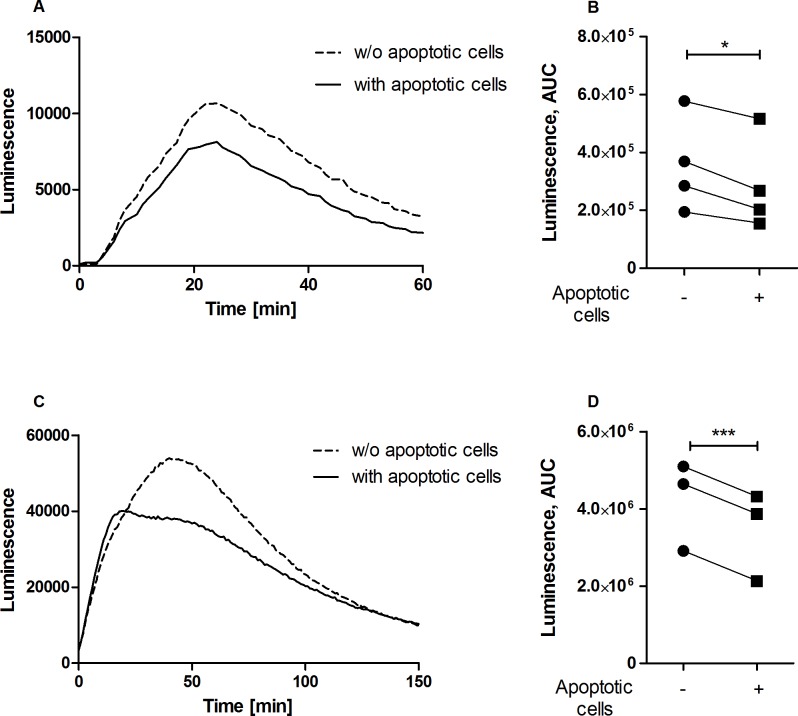
Diminished ROS production by neutrophils in the presence of apoptotic cells. ROS production was induced by co-incubation of freshly isolated neutrophils with TNF (A, B) or *L*. *major* promastigotes (C, D) in the presence or absence of apoptotic cells. ROS production was assessed by the luminol-based chemiluminescence assay. Time kinetics of ROS release (chemiluminescence) of representative experiments (A, C) and the data quantification as area under the curve (B, D; n = 3–4) are shown. * p ≤ 0.05, *** p ≤ 0.001.

The increased survival of *Leishmania* parasites in neutrophils exposed to apoptotic cells (Fig 3) suggests that antimicrobial functions are likely to be compromised by apoptotic cells. The potent leishmanicidal effector mechanism in phagocytes is generation of reactive oxygen species (ROS) [25–29]. The pro-inflammatory cytokine TNF, which is essential for protection against *L*. *major* [30,31], is a potent activator of ROS production [32–34]. TNF promotes ROS production in neutrophils via activation of the p38 mitogen-activated protein kinase (MAPK) and protein kinase (PK) Cδ signaling pathways [32,35]. Therefore, we tested whether apoptotic cells affect TNF-α-induced oxidative burst in neutrophils. Importantly, we found a markedly reduced phosphorylation of both p38-MAPK and PKCδ kinases (Fig 4) and downregulated generation of ROS in TNF-α-stimulated neutrophils after co-incubation with apoptotic cells (Fig 5A and 5B).

### Diminished ROS production by *L*. *major*-infected neutrophils in the presence of apoptotic cells

We next addressed the question whether apoptotic cells modulate generation of ROS in neutrophils infected with *Leishmania*. ROS production in neutrophils induced by *L*. *major* infection was significantly reduced when the infected neutrophils were co-incubated with apoptotic cells (Fig 5C and 5D). Together, these results support the view that uptake of apoptotic cells by *L*. *major*-infected neutrophils downregulates ROS production leading to improved survival of the parasite within neutrophils.

## Discussion

Neutrophils are the first cells that migrate to the site of *L*. *major* infection [1]. As the first line of defense, neutrophils possess numerous effector mechanisms to kill ingested pathogens [25]. However, uptake of *L*. *major* results in the suppression of effector mechanisms, allowing the survival of the parasites within neutrophils [2]. This study revealed that uptake of apoptotic cells by infected neutrophils contributes to suppression of neutrophil antimicrobial functions leading to improved survival of parasites in *L*. *major*-infected neutrophils.

We previously demonstrated that neutrophils, similarly to macrophages, can engulf apoptotic cells in vitro and this process requires the presence of normal, non-heat-inactivated, human serum [6], implying that the complement system is involved. Here we showed that the lack of the complement factors C1q and C3 in serum leads to impaired neutrophil-mediated phagocytosis of apoptotic cells. The effect was completely reversed by reconstituting C3-deficient serum with exogenous C3, whereas adding C1q to C1q-depleted serum tended to only partially restore normal phagocytosis rate, likely due to insufficient C1q concentration [14]. C1q was reported to bind to apoptotic structures such as phosphatidylserine, which is abundantly present on the surface of apoptotic cells [36], and calreticulin [37] and activates the classical complement pathway, leading to C3 deposition on the surface of apoptotic cells [38]. The products of C3 cleavage–C3b and C3bi–are recognized by the complement receptors CR1 (CD35) and CR3/4 (CD11b/CD18 and CD11c/CD18), respectively, expressed by phagocytes [14].

Our study showed that infection with *L*. *major* enhances neutrophil’s capacity to ingest apoptotic cells. To characterize this phenomenon, we firstly focused on the complement receptors known to be involved in engulfment of apoptotic cells by other professional phagocytes such as macrophages and dendritic cells [14,22]. We observed enhanced expression of CD11b (a component of CR3) and CD35 (a component of CR1), but not CD11c (a component of CR4) on *Leishmania*-infected neutrophils. Interestingly, that both CR1 and CR3 mediate *Leishmania* entry to macrophages [39,40]. In neutrophils, CR1 promotes the adhesion of bioparticles and CR3 mediates their subsequent internalization [41]. Therefore, it is reasonable to speculate that the enhanced expression of CR1 and CR3 on *Leishmania*-infected neutrophils is likely to be a strategy developed by the parasites both to accelerate the infection of phagocytes as well as to enhance uptake of apoptotic cells, which, consequently, contribute to the survival of the parasites inside neutrophils.

Along with the complement receptors, CD14 and CD44 play a role in clearance of apoptotic cells by professional phagocytes [23,24]. Surprisingly, our data revealed that the surface expression of both CD14 and CD44 was downregulated on *L*. *major*-infected neutrophils. Besides its role in apoptotic cell uptake, CD14 binds LPS and serves as a co-receptor for TLR4 [42]. It was shown that pro-inflammatory stimuli induce CD14 internalization in monocytes [43]. Since TLR2 and TLR4 are involved in the recognition of *Leishmania* by neutrophils [44], it is meaningful that infection with *L*. *major* promotes CD14 internalization in neutrophils. Further, CD44 participates in neutrophil adhesion to endothelial cells [45]. Pro-inflammatory signals, such as TNF and fMLP, lead to downregulation of CD44 expression on neutrophils to promote trafficking to inflamed tissue [46]. It is therefore likely that inflammatory activation of neutrophils during *L*. *major* infection decreases CD44 expression on neutrophil’s surface. Our data suggest that neither CD14 nor CD44 contribute to enhanced uptake of apoptotic cells by neutrophils upon *L*. *major* infection. Further studies would clarify whether, in addition to CR1 and CR3, other receptors involved in apoptotic cell uptake by neutrophils are modulated by *Leishmania* infection.

Neutrophils possess versatile molecular weaponry to kill intracellular parasites [25]. ROS production is a key defense mechanism required for destruction of *Leishmania* parasites [2,26–29]. Importantly, apoptotic cells inhibit fMLP-induced ROS production in neutrophils [6]. In the present study we demonstrated that apoptotic cells inhibit *Leishmania*-induced ROS production of neutrophils. Moreover, the ROS production induced by TNF, which is an essential mediator of immune defense against *Leishmania* [30,31], was also reduced in the presence of apoptotic cells. Signaling pathways involving the phosphorylation of PCKδ and p38 MAPK were shown to be essential for appropriate ROS generation in neutrophils [32,35,47]. We showed that co-incubation of neutrophils with apoptotic cells significantly decreased p38 MAPK and PCKδ phosphorylation and, as a consequence, ROS generation. Reduced ROS production is likely a key factor associated with enhanced survival of the parasites in neutrophil granulocytes exposed to apoptotic cells.

Recent reports suggest a role of neutrophil extracellular traps (NETs) in the killing of *Leishmania* parasites [48,49]. Since *Leishmania* can induce NETs in a ROS-dependent manner [49], the inhibition of neutrophil ROS production by apoptotic cells likely results in reduced NET release that may further facilitate the survival of *Leishmania* parasites in the infected tissue.

Previous studies showed that uptake of apoptotic cells by *Leishmania*- or *Trypanosoma*-infected macrophages leads to increased parasite burden [13,50]. Here we demonstrated that apoptotic cells exert a similar effect on *Leishmania*-infected neutrophils. It is reasonable to believe that the enhancing effect of apoptotic cells on the survival of *Leishmania* in neutrophils contributes to the parasite survival in vivo. Indeed, administration of dead neutrophils during *L*. *major* infection amplifies the parasite replication in BALB/c mice [51]. Therefore, the inhibition of phagocytosis of apoptotic cells could constitute a potential target for therapeutic interventions.

**Fig 6:**
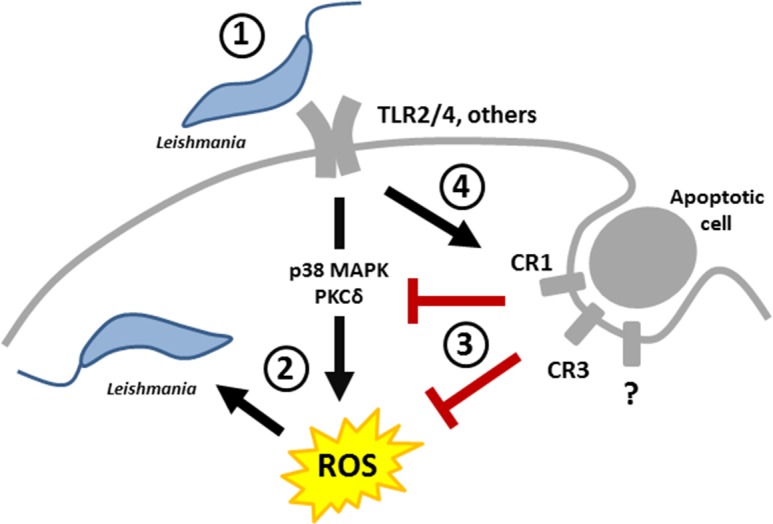
Apoptotic cells modulate the course of *L*. *major* infection in neutrophils. (1) *Leishmania* promastigotes are recognized by neutrophils; (2) *Leishmania*-infected neutrophils activate antimicrobial signaling pathways responsible for generation of ROS; (3) Signals from apoptotic cells suppress phosphorylation of pro-inflammatory kinases and inhibit ROS production in neutrophils leading to improved survival of the parasites; (4) *Leishmania* can upregulate neutrophil-mediated phagocytosis of apoptotic cells to reduce microbicidal environment within neutrophils.

*Leishmania* parasites developed different strategies to avoid destruction by phagocytes. For instance, similarly to apoptotic cells, dying *Leishmania* promastigotes in virulent inoculum expose phosphatidylserine, which serves as an anti-inflammatory signal for phagocytes [52]. Our in vitro findings suggest for the first time that *Leishmania* parasites may have an additional strategy to avoid pro-inflammatory responses by enhancing neutrophil-mediated phagocytosis of apoptotic cells, which results in decreased ROS production in *L*. *major*-infected neutrophils and thereby improved survival of the parasites (Fig 6).

## Supporting information

S1 Fig Gating strategy. Neutrophil population was gated based on cell size and granularity. Next, doublets were excluded by using SSC and FSC H & W hierarchically. After doublets discrimination phagocytosis of apoptotic cells was analyzed. Representative flow cytometry dot plots of neutrophils without (A) and with apoptotic (B) cells are shown.
(TIF)

S2 Fig Non-infected and *L. major*-infected neutrophils phagocytose apoptotic cells. Non-infected (A) and *L*. *major*-infected (B) neutrophils (green) were co-incubated with apoptotic cells (red) for 90 min in the presence of normal human serum. After co-incubation cytocentrifuge slides were prepared and analyzed by using Keyence BZ-9000 microscope. Representative micrographs are shown. Arrows indicate neutrophils that engulfed apoptotic cells.
(TIF)
