# Supplementary figures and images for: Enhanced survival of Leishmania major in neutrophil granulocytes in the presence of apoptotic cells
Source: PLoS One. 2017 Feb 10;12(2):e0171850. doi: 10.1371/journal.pone.0171850 (PMC5302790; doi:10.1371/journal.pone.0171850)

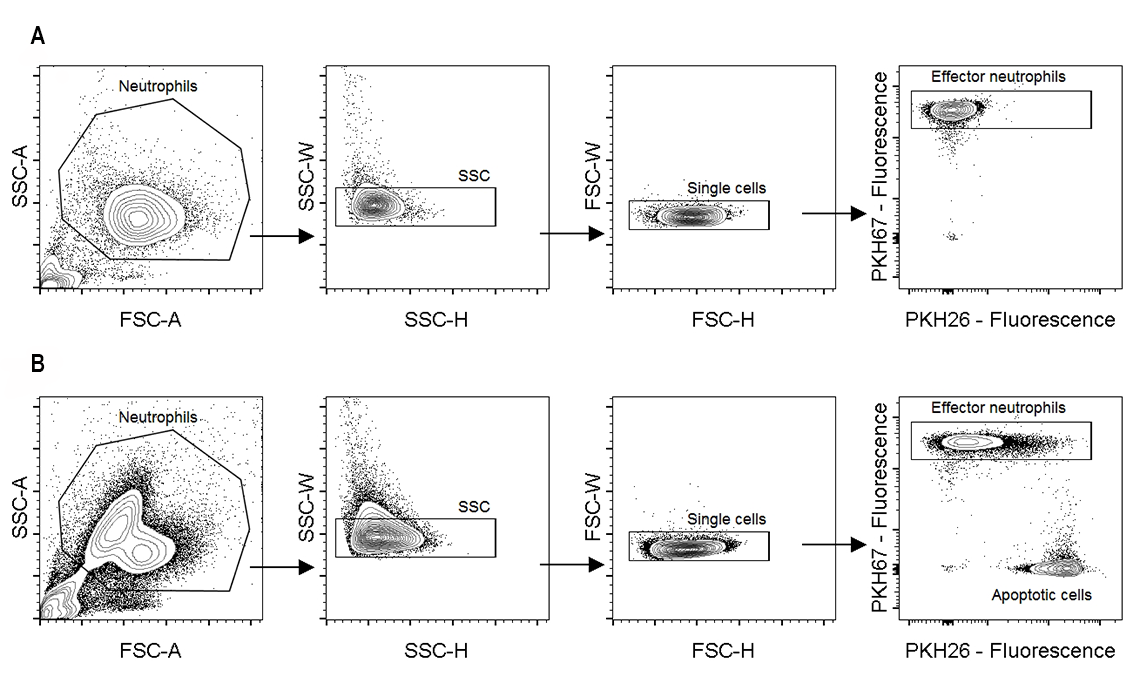

Supplement: S1 Fig — Neutrophil population was gated based on cell size and granularity. Next, doublets were excluded by using SSC and FSC H & W hierarchically. After doublets discrimination phagocytosis of apoptotic cells was analyzed. Representative flow cytometry dot plots of neutrophils without (A) and with apoptotic (B) cells are shown. (TIF) [file pone.0171850.s001.tif]

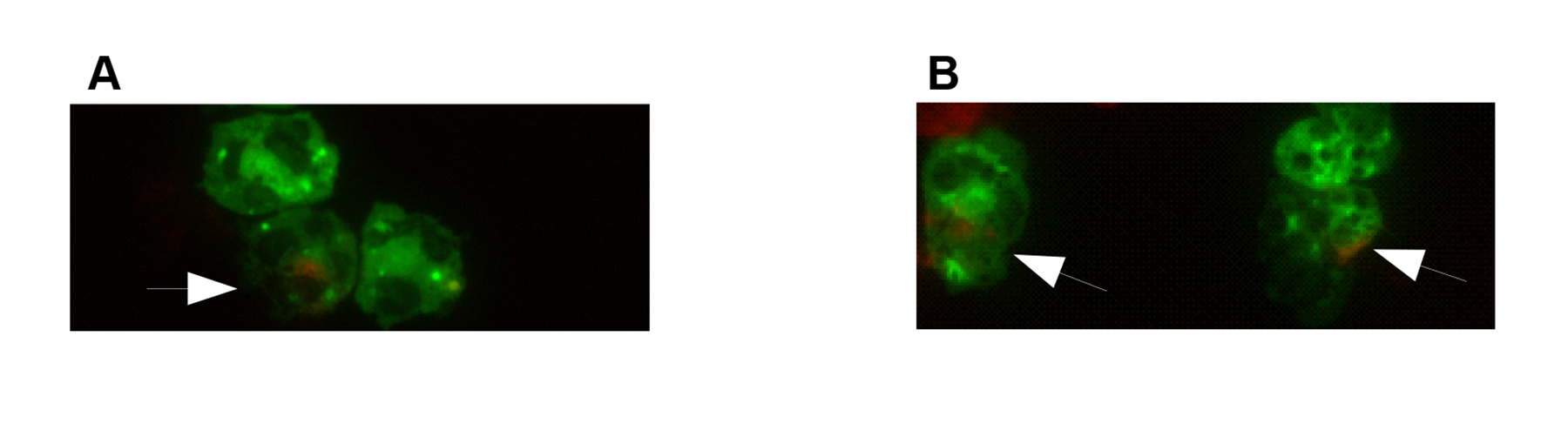

Supplement: S2 Fig — Non-infected (A) and L. major-infected (B) neutrophils (green) were co-incubated with apoptotic cells (red) for 90 min in the presence of normal human serum. After co-incubation cytocentrifuge slides were prepared and analyzed by using Keyence BZ-9000 microscope. Representative micrographs are shown. Arrows indicate neutrophils that engulfed apoptotic cells. (TIF) [file pone.0171850.s002.tif]
